# Supplementary material for: Effect of Mulberry Leaf Powder of Varying Levels on Growth Performance, Immuno-Antioxidant Status, Meat Quality and Intestinal Health in Finishing Pigs
Source: Antioxidants (Basel). 2022 Nov 14;11(11):2243. doi: 10.3390/antiox11112243 (PMC9687140; doi:10.3390/antiox11112243)
Supplement: Supplementary file 1 [file antioxidants-11-02243-s001.zip › Antioxidants Figure S1.pdf]

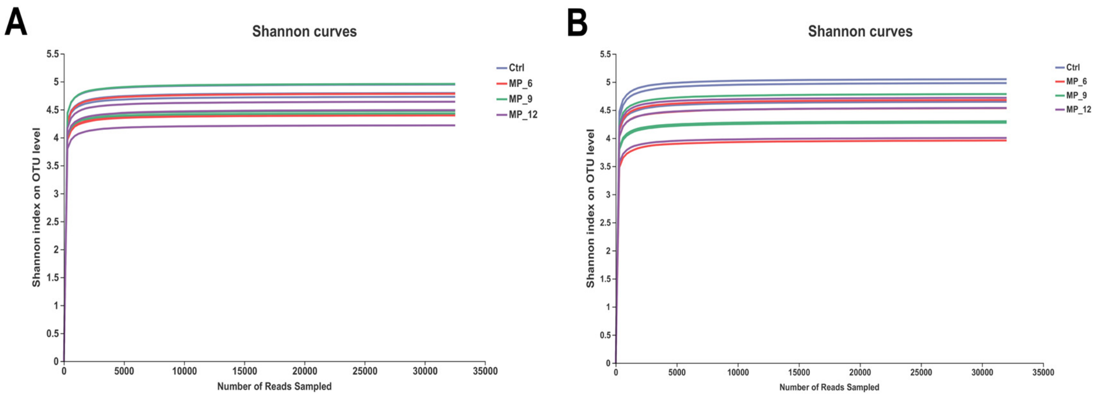

Figure S1. The rarefaction curves of cecum (A) and colon (B) at OTU level in finishing pigs dietary supplementation of mulberry leaf powder with varying levels. All samples of cecum and colon showed that the diversity indices increased sharply and eventually flatten, indicating that the sequencing data were substantial enough for reflecting the majority of microbial diversity information in the samples.
