# Supplementary material for: Effect of Mulberry Leaf Powder of Varying Levels on Growth Performance, Immuno-Antioxidant Status, Meat Quality and Intestinal Health in Finishing Pigs
Source: Antioxidants (Basel). 2022 Nov 14;11(11):2243. doi: 10.3390/antiox11112243 (PMC9687140; doi:10.3390/antiox11112243)
Supplement: Supplementary file 1 [file antioxidants-11-02243-s001.zip › Antioxidants-Figure S2.pdf]

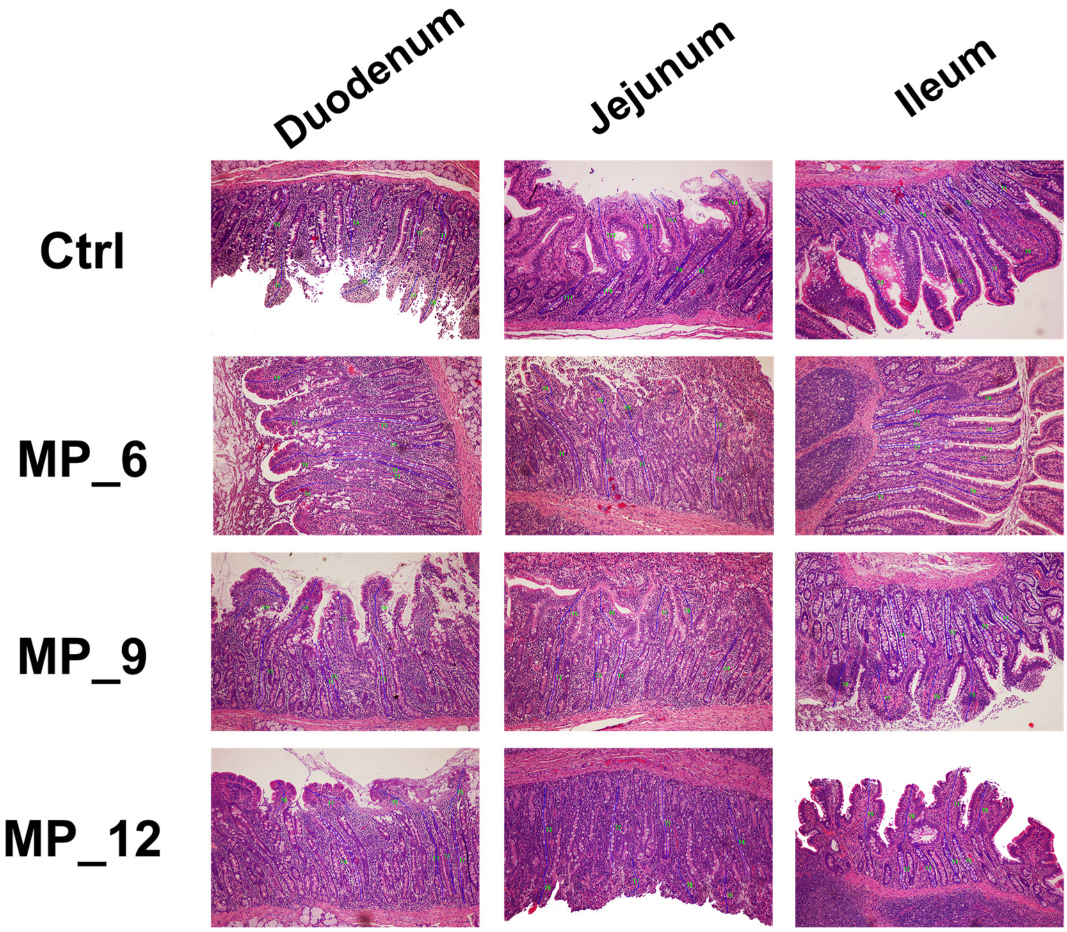

Figure S2. The photomicrograph of intestinal morphology in finishing pigs dietary supplementation of mulberry leaf powder with varying levels. Ctrl, basal diet with 0% MP; MP_6, MP_9, MP_12, the basal diet supplemented with 6%, 9% and 12% MP, respectively. The images were visualized at 100x magnification and four pairs of villi and crypt were highlighted with blue color. *n* = 3.
