# Supplementary material for: Effect of Mulberry Leaf Powder of Varying Levels on Growth Performance, Immuno-Antioxidant Status, Meat Quality and Intestinal Health in Finishing Pigs
Source: Antioxidants (Basel). 2022 Nov 14;11(11):2243. doi: 10.3390/antiox11112243 (PMC9687140; doi:10.3390/antiox11112243)
Supplement: Supplementary file 1 [file antioxidants-11-02243-s001.zip › Antioxidants-Table S1.pdf]

Table S1. Nutrient composition of mulberry leaf powder (%, as-fed basis)

Item	Mulberry powder
Dry matter	94.23
Crude protein	19.64
Ether extract	5.20
Crude fiber	34.44
Neutral detergent fiber	34.90
Acid detergent fiber	21.60
Organic matter	89.97
Gross energy	17.39
Calcium	1.64
Phosphorus	0.26
Aspartic acid	1.12
Glutamic acid	1.35
Threonine	0.51
Serine	0.46
Proline	0.49
Glycine	0.60
Alanine	0.66
Cysteine	0.07
Valine	0.64
Methionine	0.03
Isoleucine	0.51
Leucine	0.95
Tyrosine	0.32
Phenylalanine	0.58
Histidine	0.54
Lysine	0.80
Arginine	0.55
Tryptophan	0.10
