# Supplementary material for: Effect of Mulberry Leaf Powder of Varying Levels on Growth Performance, Immuno-Antioxidant Status, Meat Quality and Intestinal Health in Finishing Pigs
Source: Antioxidants (Basel). 2022 Nov 14;11(11):2243. doi: 10.3390/antiox11112243 (PMC9687140; doi:10.3390/antiox11112243)
Supplement: Supplementary file 1 [file antioxidants-11-02243-s001.zip › Antioxidants-Table S2.pdf]

Table S2 Composition and nutrient levels of basal diet (%, as-fed basis)

Item	Ctrl	6%MP	9%MP	12%MP
Corn, CP 7.6%	74.23	69.77	68.23	66.61
Soybean meal, CP 43.6%	11.78	11.16	10.60	10.12
Wheat bran, CP 17.17%	10.40	9.60	8.80	8.00
Mulberry Powder	0.00	6.00	9.00	12.00
Soybean oil	0.60	0.50	0.40	0.30
Limestone	0.93	0.92	0.90	0.87
Dicalcium phosphate	0.58	0.62	0.66	0.70
Salt	0.30	0.30	0.30	0.30
L-Lysine HCl, 78%	0.28	0.24	0.22	0.21
DL-Methionine, 98%	0.03	0.04	0.04	0.05
L-Threonine，98%	0.09	0.07	0.07	0.06
L-Tryptophan，98%	0.03	0.03	0.03	0.03
Chromic oxide	0.25	0.25	0.25	0.25
Premix[1]	0.50	0.50	0.50	0.50
Total	100.00	100.00	100.00	100.00
**Nutritional level (Caculated)**				
Digestive energy，MJ/kg	14.26	14.26	14.26	14.26
Crude protein	13.27	13.09	12.88	12.70
Lysine	0.83	0.83	0.83	0.83
Methionine	0.26	0.26	0.26	0.26
Threonine	0.57	0.57	0.57	0.57
Calcium	0.58	0.58	0.58	0.58
Total phosphorus	0.46	0.46	0.46	0.46
**Nutritional level (Analyzed)**				
Gross energy，MJ/kg	16.01	16.02	15.97	16.06
Crude protein	12.89	12.54	12.31	11.88
Ether extract	3.84	3.71	3.32	3.35
Organic matter	95.91	95.44	95.25	95.13

[1] Premix provided the following per kg of diets: vitamin A (retinyl acetate), 8,250 IU; vitamin D_3 (cholecalciferol), 825 IU; vitamin E (DL-alpha-tocopheryl acetate), 40 IU; vitamin K_3 (menadione nicotinamide bisulfite), 4.0 mg; vitamin B_1 (thiamine mononitrate), 1.0 mg; riboflavin, 5.0 mg; vitamin B_5 (DL-calcium pantothenate), 15 mg; vitamin B_6 (pyridoxine hydrochloride), 2.0 mg; vitamin

B_{12}, 25 μg; niacin, 35 mg; folacin, 2 mg; biotin, 4 mg; choline chloride, 600 mg; Mn (MnO), 25 mg; Fe ($FeSO4{\cdot}H2O$), 80 mg; Zn (ZnSO4), 100 mg; Cu ($CuSO4{\cdot}5H2O$), 50 mg; I (KI), 0.5 mg; Se (Na2SeO3), 0.15 mg
