# Supplementary material for: Effect of Mulberry Leaf Powder of Varying Levels on Growth Performance, Immuno-Antioxidant Status, Meat Quality and Intestinal Health in Finishing Pigs
Source: Antioxidants (Basel). 2022 Nov 14;11(11):2243. doi: 10.3390/antiox11112243 (PMC9687140; doi:10.3390/antiox11112243)
Supplement: Supplementary file 1 [file antioxidants-11-02243-s001.zip › Antioxidants-Table S3.pdf]

Table S3. Primer sequences of housekeeping and target genes related with intestinal tight junction and muscle antioxidant capacity

Genes	Sequences (5' - 3')	Size (bp)
Occludin	F: ATGCTTTCTCAGCCAGCGTA<br>R: AAGGTTCCATAGCCTCGGTC	176
Claudin-1	F: CAAAACCTTCGCCTTCCAG<br>R: TCCCCACATTCGAGATGATTAC	293
ZO-1	F: GAGGATGGTCACACCGTGGT<br>R: GGAGGATGCTGTTGTCTCGG	169
Mucin-2	F: CGGCTCTCCAGTCTACTCGTCTAA<br>R: TGGTTGTCGGGCAAGTTGATGA	204
SOD1	F: GAGACCTGGGCAATGTGACTG<br>R: GCCAAACGACTTCCAGCAT	190
GPX1	F: CAGGCGGCGGGTTCG<br>R: TGAGGGCAGTGGCATCGT	129
Hmox1	F: TGGTCTCTTGACTGGCTTCCTTGT<br>R: GCTTCTGGCTGGCTCCATTCTC	245
NFE2L2	F: TTCTAAGAGGTCTGCTGGCATCAC<br>R: CTGCGAGCTACACCACAGTTCAT	106
MyHC I	F: CCGAGAAGGATGAGGAGATGGAAC<br>R: ATGCGATTGGCGTGGCTGAG	175
MyHC IIa	F: ACCTACAGCACCGTCTGGATGA<br>R: GCTCACTCTCAACCTCTCCTTCC	114
MyHC IIx	F: CCTGGAGCGGATGAAGAAGAACCT<br>R: CTCGTGTTTGCGTAGACCCTTGAC	199
PGC1a	F: ACAGACTCAGACCAGTGCTACCT<br>R: GGATGACCGAAGTGCTTGTTCAG	128
Nrf2	F: ACCTGTGCCTGCTGGATTGAGA<br>R: GGTTCGTTACCACTGAGCCATAGC	162
UCP1	F: TTGTACCTTCCTTCTGGTGACTGG<br>R: CTGCCGTGACTCCATCAACTCTT	138
HSL	F: TGCGTGAAGGACAGGACAGTGA<br>R: AGGTAAGGCTCGTGGGATTTGGA	172
ChREBP	F: GGCACCCTTGGCAAACCTTTATAG<br>R: AACTCCGTTCTTCCTGGCTTCTG	136
PPARγ	F: CCAACGGCATCCAGAACAAGGAG<br>R: AACTCCGTTCTTCCTGGCTTCTG	193
β-actin	F: TACGCCAACACGGTGCTGTC<br>R: GTACTCCTGCTTGCTGATCCACAT	207

F, forward primer; R, Reverse primer; ZO-1, zonula occludens-1; SOD1, superoxide dismutase; GPX1, glutathione peroxidase 1; Hmox1, heme oxygenase 1; NFE2L2, nuclear factor erythroid 2-like 2; MyHC1, myosin heavy chain 1; MyHC IIa, myosin heavy chain IIa; MyHC IIx myosin heavy chain IIx; PGC-1α, peroxisome proliferator-activated receptor γ coactivator-1α; Nrf2, nuclear respiratory factor; UCP1, uncoupling protein-1; HSL, hormone-sensitive lipase;ChREBP, carbohydrate response element binding protein; PPARγ, peroxisome proliferator-activated receptor γ.
